# Machine Learning Methods to Estimate Individualized Treatment Effects for Use in Health Technology Assessment

**DOI:** 10.1177/0272989X241263356

**Published:** 2024-07-26

**Authors:** Yingying Zhang, Noemi Kreif, Vijay S. GC, Andrea Manca

**Affiliations:** Centre for Health Economics, University of York, UK; Centre for Health Economics, University of York, UK; Department of Pharmacy, University of Washington, Seattle, USA; School of Human and Health Sciences, University of Huddersfield, UK; Centre for Health Economics, University of York, UK

**Keywords:** machine learning, causal inference, individualized treatment effect, health technology assessment, observational data

## Abstract

**Background:**

Recent developments in causal inference and machine learning (ML) allow for the estimation of individualized treatment effects (ITEs), which reveal whether treatment effectiveness varies according to patients’ observed covariates. ITEs can be used to stratify health policy decisions according to individual characteristics and potentially achieve greater population health. Little is known about the appropriateness of available ML methods for use in health technology assessment.

**Methods:**

In this scoping review, we evaluate ML methods available for estimating ITEs, aiming to help practitioners assess their suitability in health technology assessment. We present a taxonomy of ML approaches, categorized by key challenges in health technology assessment using observational data, including handling time-varying confounding and time-to event data and quantifying uncertainty.

**Results:**

We found a wide range of algorithms for simpler settings with baseline confounding and continuous or binary outcomes. Not many ML algorithms can handle time-varying or unobserved confounding, and at the time of writing, no ML algorithm was capable of estimating ITEs for time-to-event outcomes while accounting for time-varying confounding. Many of the ML algorithms that estimate ITEs in longitudinal settings do not formally quantify uncertainty around the point estimates.

**Limitations:**

This scoping review may not cover all relevant ML methods and algorithms as they are continuously evolving.

**Conclusions:**

Existing ML methods available for ITE estimation are limited in handling important challenges posed by observational data when used for cost-effectiveness analysis, such as time-to-event outcomes, time-varying and hidden confounding, or the need to estimate sampling uncertainty around the estimates.

**Implications:**

ML methods are promising but need further development before they can be used to estimate ITEs for health technology assessments.

**Highlights:**

- Estimating individualized treatment effects (ITEs) using observational data and machine learning (ML) can support personalized treatment advice and help deliver more customized information on the effectiveness and cost-effectiveness of health technologies.
- ML methods for ITE estimation are mostly designed for handling confounding at baseline but not time-varying or unobserved confounding. The few models that account for time-varying confounding are designed for continuous or binary outcomes, not time-to-event outcomes.
- Not all ML methods for estimating ITEs can quantify the uncertainty of their predictions.
- Future work on developing ML that addresses the concerns summarized in this review is needed before these methods can be widely used in clinical and health technology assessment–like decision making.

Cost-effectiveness analysis (CEA) results are often used to inform health technology assessment adoption decisions. Several contributions have extended the standard economic evaluation framework to show that nuanced treatment and funding decisions that take into account patient characteristics may yield greater population health gains compared with one-size-fits-all policies.^1–3^ One important way in which patient characteristics can influence the value for money of a given treatment is through treatment effect heterogeneity—the fact that some individuals may gain more from a treatment than others. Learning heterogeneous treatment effects allows the identification of those patients who benefit the most (and the least) from certain treatments, facilitating stratified policy decisions.

Treatment effect heterogeneity has typically been investigated via subgroup analyses of randomized controlled trial (RCT) data using traditional statistical solutions such as treatment-by-covariate interactions in a regression model. However, these solutions yield only average treatment effects for prespecified subgroups and hence might miss important drivers of systematic variation.

A new area of research in statistics, economics, and computer science has focused on estimating treatment effect heterogeneity in a way that does not require prespecified subgroups yet yields estimates of heterogeneous treatment effects in transparent and reproducible ways. This literature aims to capture the potential complex relationship between observable patient characteristics and the expected treatment effect, often referred to as the conditional average treatment effect (CATE) function. Predictions from the CATE function can yield estimates of individualized treatment effects (ITEs). Although ITE and CATE are often used interchangeably (and we adopt this convention in our article), it is important to note that predictions of ITEs from estimated CATE functions are only individualized to the extent allowed by the richness of the observed covariate information and do not capture unobservable heterogeneity in the treatment effects.^4^

Most of this literature incorporates machine learning (ML) in a formal causal inference framework, often referred to as *causal machine learning.*^5,6^ The formal causal inference framework ensures that the sources of bias that may affect a treatment effect estimate derived from observational data, most importantly confounding, are addressed. The strengths of ML can be exploited for ITE estimation in several ways. First, ML can be used to specify so-called nuisance models (outcome regressions and propensity score models) that can help reduce the bias due to confounding in estimates of treatment effects.^7^ ML models for nuisance model estimation may be preferable to parametric models as they can data-adaptively take into account nonlinearities and interactions in the data-generating mechanism and can also select an ensemble of models to improve performance.^8^ As real-world data may be high dimensional, some ML algorithms (for example, random forests and LASSO) can also allow for selection among a large number of potential confounders.^9^ Methods that estimate ITEs can rely on these nuisance models (see more details in the “ML Methods to Estimate ITE” section) but can also flexibly characterize the relationship between observed covariates and the expected treatment effects, like the causal forests approach.^47-51^ Here, the ability of ML to perform variable selection is once again crucial as there may be only a few variables that contribute to treatment effect heterogeneity among a large number of candidates.

Applications of ML in health care have multiplied rapidly in recent years, thanks to the development of freely available estimation algorithms.^9–17^ Health economics and outcomes researchers have embraced this new approach with enthusiasm, and it is now recognized that ML is a valuable tool to capture the complexities (e.g., nonlinearity and heterogeneity) in the disease process and the costs and outcomes associated with given health states and treatments.^9^ Several published health economics and outcomes research studies have used ML to assist with, for example, selecting study population and key covariates, and a summary of these studies can be found elsewhere.^9^ However, there is little practical guidance to help health technology assessment practitioners aiming to apply ML methods specifically to estimate heterogeneous treatment effects such as ITEs. As existing ML approaches to ITE estimation have often been developed outside health care, it is not clear which (if any) of the available methods meets the needs of health technology assessment practitioners.

We use the example of migraine to illustrate the potential use of ITEs in health technology assessment. ML methods have been previously used to predict the occurrence of migraine and to identify the relevant features for prediction.^18,19^ However, when assessing the cost-effectiveness of a new migraine medication, researchers may need to go further than simple predictions and need to model the treatment-specific risk and duration of each episode as a function of an individual patient’s characteristics. Such treatment-specific risk parameters can be constructed from a baseline risk and an ITE and estimated via ML methods reviewed here. Heterogeneity can also affect the (potentially treatment-specific) cost and health-related quality-of-life parameters. A cost-effectiveness model, encapsulating these heterogeneous input parameters,^20^ can then usefully inform stratified decisions that aim to provide the right treatment to the right patient and report the value of stratification.^21^ Even when the interest is in making one-size-fits-all decisions for a predefined target population that is relevant for a given treatment, stratified model inputs can help produce more accurate cost-effectiveness analysis both for the population average and subgroup-specific results, due to the nonlinear relationship between model inputs and outputs.

This scoping review aims to identify ML methods available for estimating ITEs, for the purposes of health technology assessment decisions regarding whether payers should fund or reimburse a health technology or intervention. In the following sections, we clarify key concepts of ITE and causal inference and identify key challenges that ML methods need to overcome to be useful for estimating ITEs for use in health technology assessment, including confounding, modeling time-to-event outcomes and estimating uncertainty. We then present an intuitive overview of the currently available ML methods for ITE estimation, classifying them in terms of how they can tackle these key challenges. The article concludes by highlighting gaps and hurdles that currently hinder a more rapid adoption and successful implementation of ML for ITE estimation in health technology assessment and offers some recommendations for future research.

## Health Technology Assessment Considerations for ITE Estimation

### Challenges of Confounding in Observational Data

While RCTs are the gold standard for evaluating new health technologies, there are instances in which conducting an RCT is either unfeasible, not required for regulatory approval, or—due to strict inclusion criteria—not relevant for real-world clinical practice. In such cases, well-designed observational studies offer an alternative for nuanced estimation of comparative effectiveness and cost-effectiveness.^22–24^

**Figure 1:**
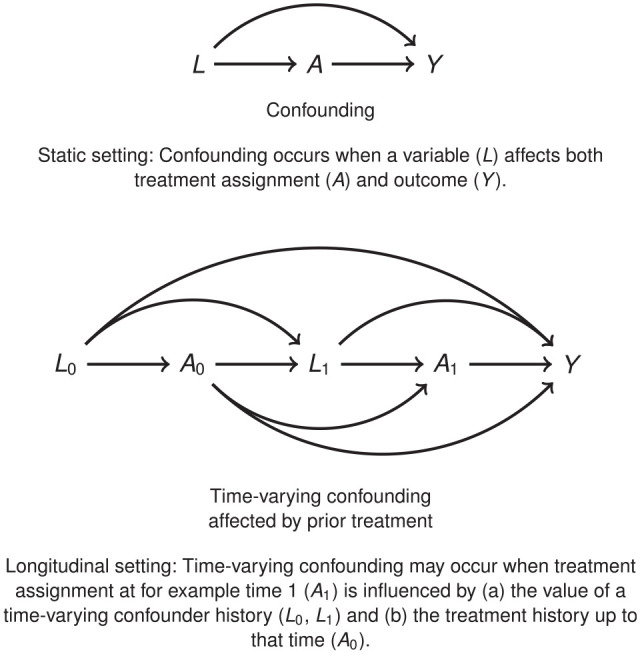
Confounding. (Top) Static setting: confounding occurs when a variable (L) affects both treatment assignment (A) and outcome (Y) . (Bottom) Time-varying confounding affected by prior treatment. Longitudinal setting: time-varying confounding may occur when treatment assignment at for example time 1 $\left(A_{1}\right)$ is influenced by (a) the value of a time-varying confounder history $\left(L_{0},\;L_{1}\right)$ and (b) the treatment history up to that time $\left(A_{0}\right)$ .^20^

To derive estimates of treatment effectiveness from observational data, the main challenge to tackle is potential bias due to confounding, as illustrated in the Directed Acyclic Graphs in Figure 1.

There is an established set of methods designed to estimate average treatment effects from observational data,^26,27^ such as regression and matching for estimating one-off treatment,^28^ inverse probability weighting estimation and g-estimation^25^ and double-robust methods^29^ for sustained treatment strategies, and instrumental variables methods for settings in which unobserved confounders cannot be excluded.^30^ While these methods do not automatically apply to estimating ITEs, the main ideas (e.g., regression adjustment in an outcome model, the weighting or double-robust correction) are exploited in causal ML estimators for ITEs. In the later sections, we discuss in detail how these can handle observed (baseline and time-varying) or even unobserved confounding.

### Estimating Relevant Parameters for Health Technology Assessment

CEA for health technology assessment often involves decision modeling.^31,32^ In this context, available data are analyzed with the aim of developing prediction models of the expected health outcomes and health care costs for a cohort of individuals, conditional on their characteristics and treatment allocation. The model will require a set of parameters to be used to make probabilistic predictions about the value of the outcomes of interest (e.g., survival time, costs, and utilities).

Some parameters capture treatment effectiveness and are expressed as treatment effects or treatment-specific mean parameters, requiring the use of causal inference methods when derived from observational data.^33^ These parameters vary depending on the outcome types and causal contrasts. For instance, quality-of-life measures may involve average treatment effects, while binary outcomes such as adverse events may require risk differences, risk ratios, or odds ratios. Time-to-event outcomes involve parameters such as differences in mean counterfactual survival times or survival probabilities. Many of these parameters can be transformed into ITE estimands by conditioning on observed characteristics (see, e.g., Hu et al.^34^ for survival outcomes). With time-to-event data, beyond the challenge of confounding, models for treatment effectiveness should also account for further potential biases from informative censoring and event-induced covariate shift where censoring or event hazard are related to individual characteristics and treatment assignment.^35,36^

### Uncertainty Quantification

The uncertainty in the input parameter values of a CEA model is a key component in the resulting decision uncertainty.^37^ Therefore, ML models used to inform treatment and funding decisions must produce a measure of uncertainty surrounding their estimates of treatment effects and predicted counterfactual outcomes. These measures of uncertainty can include standard errors, confidence intervals, or, in the case of Bayesian techniques, credible intervals of posterior distributions. Probabilistic sensitivity analysis can then be used to propagate the uncertainty in the CEA input parameters through the model and to assess their effects on decision uncertainty.

## ITE Defined

We first illustrate the counterfactual reasoning necessary to conceptualize ITE in Table 1. The treatment received is represented by a binary variable A , which takes value 1 if the subject receives treatment and 0 if not. For simplicity, we define a binary outcome variable Y , taking value 1 if the event of interest occurs and 0 otherwise, but the setting holds more generally. For each subject, we have access to a set of covariates X .

**Table 1:** Illustrating the Fundamental Problem of Causal Inference

ID	A	Y	X	$Y^{a=0}$	$Y^{a=1}$	$\mathrm{ITE}(x_{i})$
1	1	1	$x_{1}$	**0**	1	1
2	1	0	$x_{2}$	**0**	0	0
…	…	…	…	…	…	…
n-2	0	1	$x_{n-2}$	1	**0**	−1
n-1	1	0	$x_{n-1}$	**1**	0	−1
n	0	0	$x_{n}$	0	**0**	0

*A*, treatment variable (0 = no treatment; 1 = treatment); *Y*, outcome variable (0 = no event; 1 = event); *X*, baseline covariates; $Y^{a=0}$ and $Y^{a=1}$ , (potential) outcomes that would have been observed under treatment values *a* = 0 and *a* = 1, respectively. To indicate that we can observe the outcome only under the treatment the subject actually received, the counterfactual is represented in bold font.

We assume that for each subject we observe their outcomes under each alternative exposure level, that is, their potential outcomes^38,39^: $Y^{a=1}$ is the outcome that would have been observed under treatment value a=1 and $Y^{a=0}$ is the outcome that would have been observed under treatment value a=0 .

The true treatment effect for individual i is defined as $Y_{i}^{a=1}-Y_{i}^{a=0}$ . The average treatment effect (ATE) is defined as the population average of these individual differences, $E[Y_{i}^{a=1}-Y_{i}^{a=0}]$ , while the conditional average treatment effect (CATE) is defined as the expected difference in the potential outcomes, for a specific profile of covariate values $X_{i}=x$ :

(1) $$\mathrm{ITE}(x)=E[Y_{i}^{a=1}-Y_{i}^{a=0}|X_{i}=x]$$

While the economics and statistics literature refers to the above quantity as CATE,^6^ this article adopts the language of ITE from the causal ML community^40^ to highlight the fact that the resulting estimates can potentially be very granular, individualized to the extent of the observable information.

We also note that the ITE(x) above is defined for the setting of one-off binary treatment with baseline confounding, in which an additive causal contrast is of interest. The methods reviewed in the next section for more complex settings may modify and extend this estimand,^34^ but due to space constraints, we restricted our illustration to the simple case of the ITE(x) .

Due to the fundamental problem of causal inference, only one potential outcome can be observed at any given time, and therefore, ITE(x) cannot be derived without further assumptions: consistency, conditional exchangeability, positivity, and no interference (see Table 2).

**Table 2:** Identifiability Assumptions

Consistency	The consistency assumption implies that an individual’s potential outcome under observed exposure history is the outcome that will actually be observed for that person. With a dichotomous treatment A=(0,1) , consistency can be expressed as $Y=AY^{a=1}+(1-A)Y^{a=0}$ .
Conditional exchangeability	The potential outcomes are independent of treatment assignment, conditional on a set of observed covariates $X_{i}$ . In randomized controlled trials, exchangeability holds unconditionally.
Positivity	Each subject should have a nonzero probability of either treatment assignment.
No interference	The potential outcomes for one subject do not depend on the treatment assignment of others.

When these assumptions hold, they allow us to reexpress the ITE(x) in terms of observed variables only^41^:

(2) $$\begin{array}{c} \mathrm{ITE}(x)=E[Y_{i}^{a=1}-Y_{i}^{a=0}|X_{i}=x]\\ =E[Y_{i}^{a=1}|X_{i}=x]-E[Y_{i}^{a=0}|X_{i}=x]\\ =E[Y_{i}^{a=1}|A=1,X_{i}=x]-E[Y_{i}^{a=0}|A=0,X_{i}=x]\\ =E[Y_{i}|A=1,X_{i}=x]-E[Y_{i}|A=0,X_{i}=x] \end{array}$$

With a sufficiently large data set, one could estimate this quantity by finding matched pairs for each covariate value combination $X_{i}=x$ of interest, and the difference in the observed outcomes for these pairs could be interpreted as a nonparametric estimate of ITE(x) . Such an approach has two drawbacks. First, in practice, analysts work with limited data sets providing an insufficient number of treatment-control pairs, if the covariate vector of interest is more complex than a few categorical variables. Furthermore, such analysis would be prone to overfitting; that is, ITEs estimated in one data set would not be a good characterization of treatment effects in a different sample of the same population.

To overcome these problems, a wide range of ML methods have been proposed in the literature to estimate ITE(x) . Some methodological approaches derive the ITE(x) by first generating predictions for both potential outcomes, via flexible modeling of the outcome regression, and constructing the ITE as a difference in predicted potential outcomes $E[Y_{i}^{a=1}|X_{i}=x]$ and $E[Y_{i}^{a=0}|X_{i}=x]$ . Other approaches also involve further nuisance models, such as propensity scores, and may directly target the estimation of the ITE(x) as opposed to generating predictions of the potential outcomes.

In this review, we focus on methods that aim to estimate ITEs, and we note whether they also generate predicted potential outcomes. Predicted potential outcomes play an important role in health technology assessment, as they capture treatment-specific mean parameters for a given covariate profile. The related literature of counterfactual predictions focuses on generating such predictions,^42^ and we briefly refer to it in the “Discussion” section.

## ML Methods to Estimate ITE

This section provides an intuitive summary of the currently available methods in the statistical, causal inference, and computer science literature for estimating ITEs.

We direct readers who are unacquainted with ML methods to explore informative tutorials or introductory articles that elucidate the utilization of ML methods in health care and health economics.^40,43,44^ It is important to note that the article does not encompass all limitations associated with ML methods. The Professional Society for Health Economics and Outcomes Research (ISPOR)’s ML Methods Emerging Good Practices Task Force has published comprehensive guidance addressing the use of ML in health economics and outcomes research and decision making.^9^

To identify the relevant ML methods, we use the citation pearl searching method,^45^ asking experts for key articles in this area and integrating these with two key reviews: Bica et al.^40^ reviewed ML methods for ITEs for a clinical and computer science audience, while Jacob^44^ considered them from an econometric perspective.

We thoroughly examined the references and citations of these review articles and used search engines (such as Google Scholar) to search for the latest advancements in relevant ML methods. We note that the literature on optimal policy learning and dynamic treatment regimes^46–48^ was out of scope for this review, as they are concerned with finding the individualized treatment rule with the largest expected benefits, and while they may use estimates of ITEs, generating these is not their main focus.

We then developed a taxonomy that organized the methods reviewed to address the key challenges in health technology assessment. This taxonomy was shared with a group of 20 health economists specializing in health technology assessment methods, who provided valuable feedback on its content and structure.

In our final taxonomy (see Tables 3 and 4), we focus on the following challenges for health technology assessment: 1) whether the ML methods address confounding, in particular time-varying confounding and unobserved confounding; 2) whether the ML methods can be used to estimate ITEs on time-to-event outcomes; 3) whether the ML methods produce uncertainty estimates, and for what kinds of outputs (predicted potential outcomes or estimated treatment effects or both). We also group the ML methods based on the settings they deal with (static or longitudinal). Finally, we provide information about the accessibility of ML methods.

**Figure 2:**
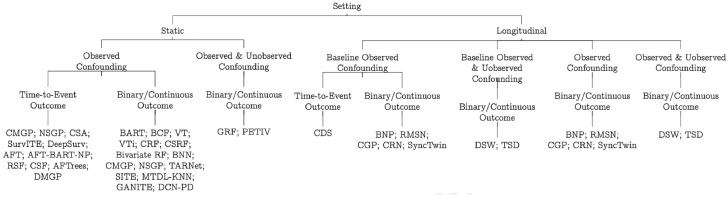
A taxonomy of statistical and machine learning individualized treatment effects estimation methods for use in health technology assessment. AFT, non-parametric accelerated failure time models; AFT-BART-NP, nonparametric Bayesian additive regression trees within the framework of accelerated failure time model; BART, Bayesian additive regression trees; BCF, Bayesian causal forest; BNN, balancing neural network; BNP, Bayesian nonparametric method; BRF, bivariate random forest; BTRC, Bayesian treatment response curves; CDS, causal dynamic survival model; CF, causal forest; CGP, counterfactual Gaussian process; CMGP, causal multitask Gaussian processes; CRF, counterfactual random forest; CRN, counterfactual recurrent network; CSA, counterfactual survival analysis; CSF, causal survival forest; CSRF, counterfactual synthetic RF; DeepSurv, Cox proportional hazards deep neural network; DMGP, deep multitask Gaussian processes; DCN-PD, deep counterfactual networks with propensity dropout; DSW, deep sequential weighting; GANITE, generative adversarial nets for inference of individualized treatment effects; MTDL-KNN, multitask deep learning and K-nearest neighbors; NSGP, nonstationary Gaussian processes; PETIV, person-centered treatment effects using a local instrumental variables; RMSN, recurrent marginal structural networks; RSF, random survival forests; SITE, local similarity preserved individual treatment effect; SurvITE, individualized treatment effect estimator for survival analysis; TARNet, treatment-agnostic representation network; TSD, time series deconfounder; VT, virtual twins random forests; VTi, virtual twins interaction models.

In Figure 2, we categorize all of the ML techniques reviewed in this article, using a tree-based diagram, following the taxonomy outlined above. Readers interested in reading these ML articles can go to the study by Bi et al.^79^ first, as it summarizes some commonly used ML terms and their equivalent terms in epidemiology. We structure our review into three parts: methods applicable for static settings, those handling longitudinal settings, and methods that can handle time-to-event outcomes, for both static and dynamic settings.

### Static Settings

In a static setting, the aim is to estimate the effect of one-time treatment decisions using data collected once (so-called cross-sectional data) or data where baseline confounders, a treatment variable, and the outcome are measured only once. When using observational data, confounding according to baseline characteristics may be present and needs to be dealt with.

We report the key features of the available ML methods to estimate ITE in a static setting in Table 3. These ML algorithms differ in the way they handle observed confounding. Some control directly for covariates (e.g., Bayesian additive regression trees, random forests), some flexibly control for the propensity score (e.g., Bayesian additive regression trees,^80^ Bayesian causal forest,^10^) while deep counterfactual networks with propensity dropout^60^ and nonstationary Gaussian processes^53^ use a doubly robust approach in which they estimate a propensity function and an outcome model using a deep multitask network or Bayesian nonparametric methods. Causal multitask Gaussian processes^52^ use a Bayesian approach to learn about the unobserved counterfactual outcomes and take into account the uncertainty in counterfactual outcomes without explicitly modeling the propensity score.

**Table 3:** Methods to Estimate Individualized Treatment Effect in Static Settings

Method	Confounding	Outcome	Uncertainty	Software
ML for continuous and binary outcomes				
Bayesian additive regression trees,^49–51^ Bayesian causal forest^10^	O	B, C	UoT, UoP	R: BART, bart-Cause, bcf
Causal forest,^10–14^ causal multitask Gaussian processes,^52^ nonstationary Gaussian processes^53^	O	B, C	UoT, UoP	R: random-Forest-SRC, grf, BayesTree, causal-Forest
Virtual twins random forests, virtual twins interaction models, counterfactual random forest, counterfactual synthetic, bivariate random forest^54–56^	O	B, C	UoT, UoP	R: aVirtualTwins, model4you
Balancing neural network^57^	O	B, C	No	No
Treatment-agnostic representation network^58^	O	B, C	UoT	Python: cfrnet
Local similarity preserved individual treatment effect^59^	O	B, C	No	Python: SITE
Deep counterfactual networks with propensity dropout^60^	O	B, C	UoT	Python: DCN-PD
Multitask deep learning and K-nearest neighbors^61^	O	B, C	No	Python: CNN
Generative adversarial nets for inference of individualized treatment effects^62^	O	B, C	UoP	Python: GANITE
Person-centered treatment effects using a local instrumental variables^63^	O, U	B, C	UoT, UoP	Stata: petiv
ML for time-to-event outcomes				
Counterfactual survival analysis^36^	O	TTE	UoT, UoP	Python: CSA
Individualized treatment effect estimator for survival analysis (SurvITE)^35^	O	TTE	No	Python: SurvITE
Cox proportional hazards deep neural network (DeepSurv)^64^	No	TTE	UoP	Python: DeepSurv
Nonparametric accelerated failure time models^64,65^	O	TTE	UoT, UoP	R: AFTrees
Nonparametric Bayesian additive regression trees within the framework of accelerated failure time model^34^	O	TTE	UoT, UoP	R: AFTBART-NP
Random survival forests^66–68^	O	TTE	UoT	No
Causal survival forest^66^	O	TTE	UoT	R: grf
Deep multitask Gaussian processes^52,53,69^	O	TTE	UoT, UoP	Python: DMGP

B, binary outcome; C, continuous outcome, O, observed confounding; ML, machine learning; TTE, time-to-event outcome; U, unobserved confounding; UoP, produces uncertainty estimates of predicted counterfactual outcomes; UoT, produces uncertainty estimates of treatment effect estimates.

The balancing neural network approach^57^ and treatment-agnostic representation network^58^ use representation learning, a process that encourages similarity between the treated and control populations. The approach of local similarity preserved individual treatment effect^59^ not only balances the distributions of control and treated groups but also uses information on local similarity—akin to nearest neighbor methods—that provides meaningful constraints on the ITE estimation.

Many of the algorithms reported in Table 3 have been designed for binary or continuous outcomes. Those methods that have been extended for use with time-to-event data are summarized in the subsection “Time-to-Event Outcomes.” The nonstationary Gaussian processes^53^ approach performs well in regimes of both small and large samples.

Methods that account for the uncertainty of treatment effect estimates include all forest-based models and deep counterfactual networks with propensity dropout. The approach of generative adversarial nets for inference of individualized treatment effects^62^ only provides uncertainty for the counterfactual outcomes. Few approaches provide uncertainty estimates for both the counterfactual outcomes and treatment effect estimates, including Bayesian additive regression trees, causal multitask Gaussian processes, and nonstationary Gaussian processes. The approaches of balancing neural network, local similarity preserved individual treatment effect, and multitask deep learning and K-nearest neighbours do not provide uncertainty quantification at all.

None of the ML methods reviewed in this section deal with unobserved confounding. Hence, we consider an alternative, parametric approach to estimate ITEs when unobserved confounding cannot be ruled out, the method of person-centered treatment effects using local instrumental variables (IV).^63^ The method, implemented as a Stata package,^81^ can be used for continuous, binary, or count data. A simplified version for continuous outcomes has been developed by Zhou and Xie^82^ for estimation in R. ML can also be applied to learn the local average treatment effect in an IV setting,^83,84^ as the first stage of a linear instrumental variables regression is effectively prediction. ML IV may perform better than non-ML IV because they are better at prediction. Nonetheless, if the ML method does not produce uncertainty estimates, it is of no use in health technology assessment. Besides IV, traditional methods also use panel data and fixed-effects or random-effects models to control for unobserved confounding, and we will discuss in the next section how ML methods deal with unobserved confounding in longitudinal settings.

### Longitudinal Settings

In a longitudinal setting, a sequence of treatment decisions and treatment effects is studied, using data collected repeatedly, such as longitudinal electronic health records or registry data. In such settings, for example when evaluating interventions for chronic conditions, treatment exposure may change over time, with decisions to start, discontinue, or switch treatment depending on the changing prognosis of the patient. Estimating relevant measures of treatment effects necessitates controlling for time-varying confounding (see Figure 1).

Most of the existing ML methods for longitudinal data, as shown in Table 4, make the unconfoundedness assumption (“sequential randomization”) that at each time step, all the past variables affecting the patient’s treatment and outcomes are observed. The Bayesian nonparametric method^70^ can predict counterfactual outcomes and estimate individualized treatment response in a continuous-time trajectory. The Bayesian treatment response curves approach^71^ extends the previous method to model multivariate outcomes, albeit not being able to handle confounding. The counterfactual Gaussian process method^72^ can both predict counterfactual outcomes and estimate ITEs on a continuous-time trajectory while accounting for baseline and time-varying confounding. The recurrent marginal structural networks approach^73^ accounts for time-varying confounding using inverse probability of treatment weighting and predicts time-dependent counterfactual outcomes using deep learning. The counterfactual recurrent networks approach^74^ accounts for time-varying confounding using representation learning that, at each time step, breaks the association between patient history and treatment assignment.

**Table 4:** Methods to Estimate Individualized Treatment Effect i n Longitudinal Settings

Method	Confounding				
	Baseline	Time Varying	Outcome	Uncertainty	Software
ML for continuous and binary outcomes					
Bayesian nonparametric method^70^	O	O	C	UoP	No
Bayesian treatment response curves^71^	No	No	C	UoP	No
Counterfactual Gaussian process^72^	O	O	C	UoP	No
Recurrent marginal structural networks^73^	O	O	B, C	No	Python: RMSN
Counterfactual recurrent network^74^	O	O	B, C	No	Python: CRN
Deep sequential weighting^75^	O, U	O, U	C	No	Python: DSW
SyncTwin^76^	O	O	C	No	Python: synth control
Time series deconfounder^77^	O, U	O, U	B, C	No	Python: TimeSeries -Deconfounder
ML for time-to-event outcomes					
Causal dynamic survival model^78^	O	No	TTE	UoT, UoP	Python: CDS

B, binary outcome; C, continuous outcome, ML, machine learning; O, observed confounding; TTE, time-to-event outcome; U, unobserved confounding; UoP, produces uncertainty estimates of predicted counterfactual outcomes; UoT, produces uncertainty estimates of treatment effect estimates.

Only two ML methods^75,77^ account for unobserved confounders. The time series deconfounder approach takes advantage of the dependencies between the multiple treatment assignments and estimates a factor model to capture the distribution of assigned treatments, using histories of covariates and treatment assignments.^77^ The sequence of latent variables is used to adjust for bias due to unobserved confounding. The deep sequential weighting approach infers the unobserved confounders using a deep recurrent weighting neural network that leverages the currently observed covariates and previous covariates and treatment assignments and computes the time-varying inverse probability of treatment for each individual to balance the confounders. The learned representations, a process in which ML algorithms are used to extract meaningful patterns from raw data to create representations of the latter that are easier to understand and process for analysis purposes, of hidden confounders and observed covariates are then combined together to predict the required potential outcomes.^75^ The SyncTwin approach^76^ uses a unique verification procedure to assess the presence of unobserved confounders. While it cannot control for unobserved confounding, it offers insights into the magnitude of the unobserved confounding problem by assessing the potential impact of unobserved confounders on pretreatment outcomes.

Estimating uncertainty in longitudinal settings becomes more intricate due to the need to estimate not only the individual-level random error at each time point but also the time-dependent random error specific to a particular treatment type. Of the methods reported in Table 4, only the Bayesian nonparametric method, the Bayesian treatment response curves, and the counterfactual Gaussian process approach can quantify uncertainty around the estimates.

### Time-to-Event Outcomes

Time-to-event outcomes such as progression-free survival or overall survival are of key interest for health technology assessment. Our review found that ML methods for estimating ITE on time-to-event outcomes are sparse (see Tables 3 and 4, “ML for time-to-event outcomes”). Among them, counterfactual survival analysis^36^ can estimate ITEs with nonparametric uncertainty quantification. The individualized treatment effect estimator for survival analysis (SurvITE)^35^ estimates treatment-specific hazard and survival functions but does not calculate uncertainty and assumes random censoring. The Cox proportional hazards deep neural network (DeepSurv)^64^ models interactions between a patient’s covariates and treatment using a neural network and produces confidence intervals for the predicted counterfactual outcomes. The nonparametric accelerated failure time approach^65^ extends Bayesian additive regression trees to survival outcomes but does not account for informative censoring. A further development is the approach of nonparametric Bayesian additive regression trees within the framework of accelerated failure time,^34^ which fits two survival outcome regression models to two sets of the observed data (one for treatment and one for control groups) and produces counterfactual survival curves conditional on individual covariate profiles. This method can also account for covariate-dependent censoring given baseline covariates. Both nonparametric accelerated failure time models and nonparametric Bayesian additive regression trees produce estimates of standard errors and uncertainty intervals for the regression coefficients.

The random survival forest methods^67,68^ have been used to estimate ITEs, assuming unconfoundedness conditional on the baseline covariates and random censoring. The causal survival forest^66^ approach adapts the causal forest algorithm^13^ and adjusts for censoring using doubly robust estimation. Modeling competing risks is another challenge in estimating ITE for time-to-event data. Deep multitask Gaussian processes^52,53,69^ can be used for survival analysis with competing risks and produces patient-specific and cause-specific survival curves with uncertainty estimates. Nonetheless, the above methods handle confounding only at baseline.

For use with longitudinal data and time-to-event outcomes, the causal dynamic survival model^78^ is the first to estimate sequential treatment effects on time-to-event outcomes in the presence of time-varying covariates. Nonetheless, this approach does not account for time-varying confounders or unobserved confounding.

## Discussion

This article provides an overview of existing ML algorithms for estimating ITEs using real-world data, for the purposes of assessing them in relation to their suitability for use in the context of health technology assessment to support more nuanced treatment and funding decisions. We find two major areas in which existing ML methods do not yet meet the needs of data analysis for health technology assessment.

First, real-world data used for health technology assessment are often longitudinal, with concerns of time-varying confounding and handling time-to-event data to derive effectiveness. The few ML methods that can estimate ITEs while accounting for time-varying confounding cannot currently handle time-to-event data. Issues of informative censoring and event-induced covariate shift make estimating ITEs technically more challenging in the context of real-world time-to-event data analysis.

Second, many of the ML algorithms this article discussed do not quantify uncertainty surrounding the ITEs or potential outcomes predictions, especially ML methods developed for longitudinal settings. The ability to produce appropriate measures of uncertainty should be a key consideration when selecting among methods.^21^ Analysts are also encouraged to use more than one method to assess the robustness of the results and consult published simulation evidence to assess the strength and weaknesses of different methods.

To ensure the acceptability of causal effects estimated using real-world data and ML for regulators and decision makers, it is crucial to evaluate the assumptions underlying specific models. The unconfoundedness assumption requires informed judgments rooted in domain expertise, usually supported by covariates-rich data sets. The overlap assumption necessitates empirical validation. Although this article focuses on the challenges of real-world data, the methods reviewed can also use RCT data to estimate ITEs.^85^

While data-driven approaches such as ML can help to arrive at a flexible yet parsimonious model, they are not substitutes for content knowledge and clinicians’ opinions. Researchers should not choose variables purely based on their performance in the model.^26^ Clinicians’ insights are important in discerning which patient characteristics influence treatment decisions and responses, and they play a pivotal role in validating a model’s treatment effects or potential outcomes estimates.^86,87^

Two related strands of the methodological literature have made progress in solving some of the challenges identified. First, causal inference methods that can account for time-varying confounding and handle time-to-event outcomes such as the longitudinal targeted minimum loss-based estimation method (LTMLE)^88^ can benefit from ML to improve model specification (see, e.g., Schomaker et al.^89^). However, these methods estimate average treatment effects, not ITEs, and are thus not reviewed here. Second, the optimal treatment regimes (in static settings) and optimal dynamic treatment regimes (in longitudinal settings)^90,91^ methods have similar goals compared with estimating ITEs, which is tailoring the right treatment to the right individual. Some of these methods^47^ use estimates of ITEs to make the treatment allocation decisions, while others, such as outcome weighted learning^92^ and dynamic weighted ordinary least squares,^93^ search for the optimal allocation without estimating ITEs. A key difference with the methods reviewed here is that the optimal treatment rules focus on the expected ATE of administering a given individualized treatment rule rather than predictions of the ITEs or counterfactual outcomes.

The policy implications of more granular cost-effectiveness results are that policy makers can appreciate the tradeoff resulting from a one-size-fits-all reimbursement decision versus one that allows reimbursing different interventions for different subgroups. Even when the interest is in making population average decisions, there is still a need to predict the prognosis, costs, and health-related quality of life more accurately for individuals by taking into account factors that truly affect these outcomes.^2,3,94^

## Recommendations for Future Research

Our article highlighted the dearth of options to estimate ITEs when there is longitudinal data with time-varying confounding. The emerging literature on ML methods for counterfactual prediction, or predictions under hypothetical interventions (details can be found in the scoping review of Lin et al.^42^) is promising in this setting and has a potential for augmenting the health technology assessment toolbox. These methods aim to estimate predicted outcomes of individuals who were to follow a particular treatment strategy, given their individual characteristics, and can produce ITEs by taking differences of counterfactual predictions under different hypothetical interventions. However, challenges such as how to validate the counterfactual prediction models or how to estimate uncertainty are currently unresolved.^95^

This article did not consider the challenges of estimating popular measures of relative treatment effects for survival data, such as the hazard ratios, as Hernán^96^ points out that hazard ratios are not an ideal treatment effect estimate for causal inference because of the sensitivity to the duration of follow-up and the inherent selection bias in period-specific hazard ratios. Nonetheless, hazard ratios can be produced by directly modeling counterfactual outcomes in a time-to-event process and transforming the counterfactual survival probabilities to hazard ratios.

Future work on developing ML methods that address the concerns summarized in this review is needed before they can be widely used in clinical and health technology assessment–like decision making. Cross-disciplinary collaboration between health science and computer science, and involving researchers as well as regulators, can accelerate the process.

## Conclusions

More work needs to be done for ML methods to become an established health economics and outcomes research tool. Researchers should focus on developing existing and new algorithms that deal with the typical data structures analyzed in health economics and outcomes research for health technology assessment and that produce the types of output required to inform individualized decisions. Programmers should try and develop more accessible software packages and tutorials to facilitate the application of the methods. Licensing and reimbursement authorities should make their position clear with regard to the role and use of evidence derived from real-world data for their decision making.
